# The effects of hookah/waterpipe smoking on general health and the cardiovascular system

**DOI:** 10.1186/s12199-019-0811-y

**Published:** 2019-09-14

**Authors:** Hanan Qasim, Ahmed B. Alarabi, Karem H. Alzoubi, Zubair A. Karim, Fatima Z. Alshbool, Fadi T. Khasawneh

**Affiliations:** 1 Department of Pharmaceutical Sciences, School of Pharmacy, The University of Texas at El Paso, El Paso, Texas 79902 USA; 2 Department of Clinical Pharmacy, Jordan University of Science and Technology, Irbid, Jordan

**Keywords:** Hookah, Waterpipe, Smoking, Cardiovascular disease, Toxicity

## Abstract

Hookah or waterpipe smoking or use is an emerging trend in the US population, especially among the youth. The misperception of hookah being less harmful than cigarettes and the availability of different but “appealing” flavors are considered among the main reasons for this trend. Hookah users however are exposed to many of the same toxic compounds/by-products as cigarette users, but at dramatically higher levels, which might lead to more severe negative health effects. In fact, hookah users are at risks of infections, cancers, lung disease, and other medical conditions. Moreover, because of the overlapping toxicant/chemical profile to conventional cigarettes, hookah smoke effects on the cardiovascular system are thought to be comparable to those of conventional cigarettes. A major source of tobacco addiction is nicotine, whose levels in hookah are extremely variable as they depend on the type of tobacco used. Taken together, in this review of literature, we will provide insights on the negative health effects of hookah in general, with a focus on what is known regarding its impact on the cardiovascular system.

## Introduction

**Fig. 1:**
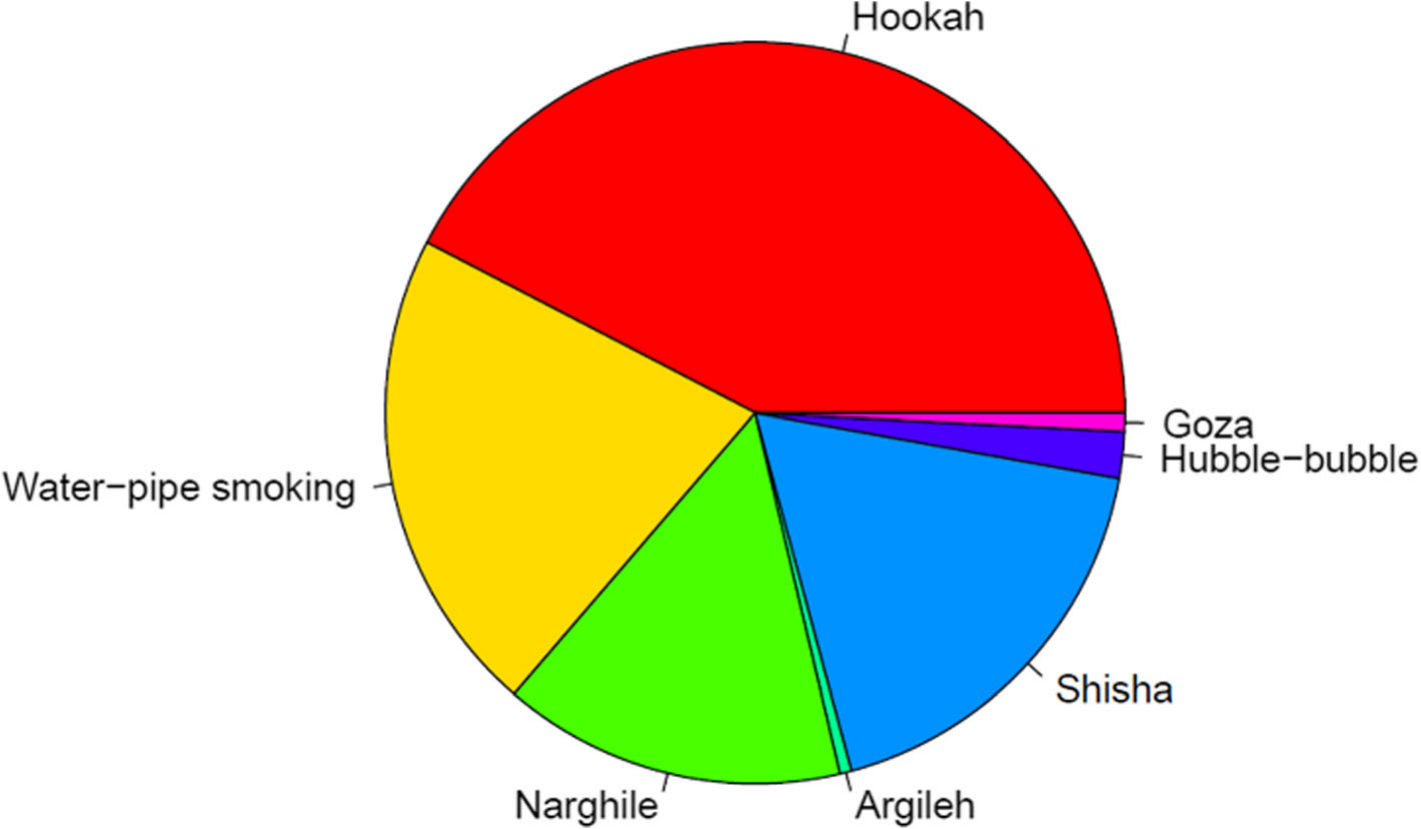
PubMed search results for different names of hookah used in the literatures. Hookah is the most common used term followed by waterpipe and shisha

Hookah also known as waterpipe, narghile, argileh, shisha, hubble-bubble, goza, borry, qaylan, chica, and mada’a (Fig. 1) is a tobacco pipe with a long yet flexible tube that draws the smoke through water contained in a bowl [1]. Even though hookah use in the western world is a recent trend, it has existed for a millennium, emerging in the North Western provinces of India, spreading to Iran, the Arab world, and Turkey and now gaining popularity in the USA and Europe [2].

### Apparatus description

**Fig. 2:**
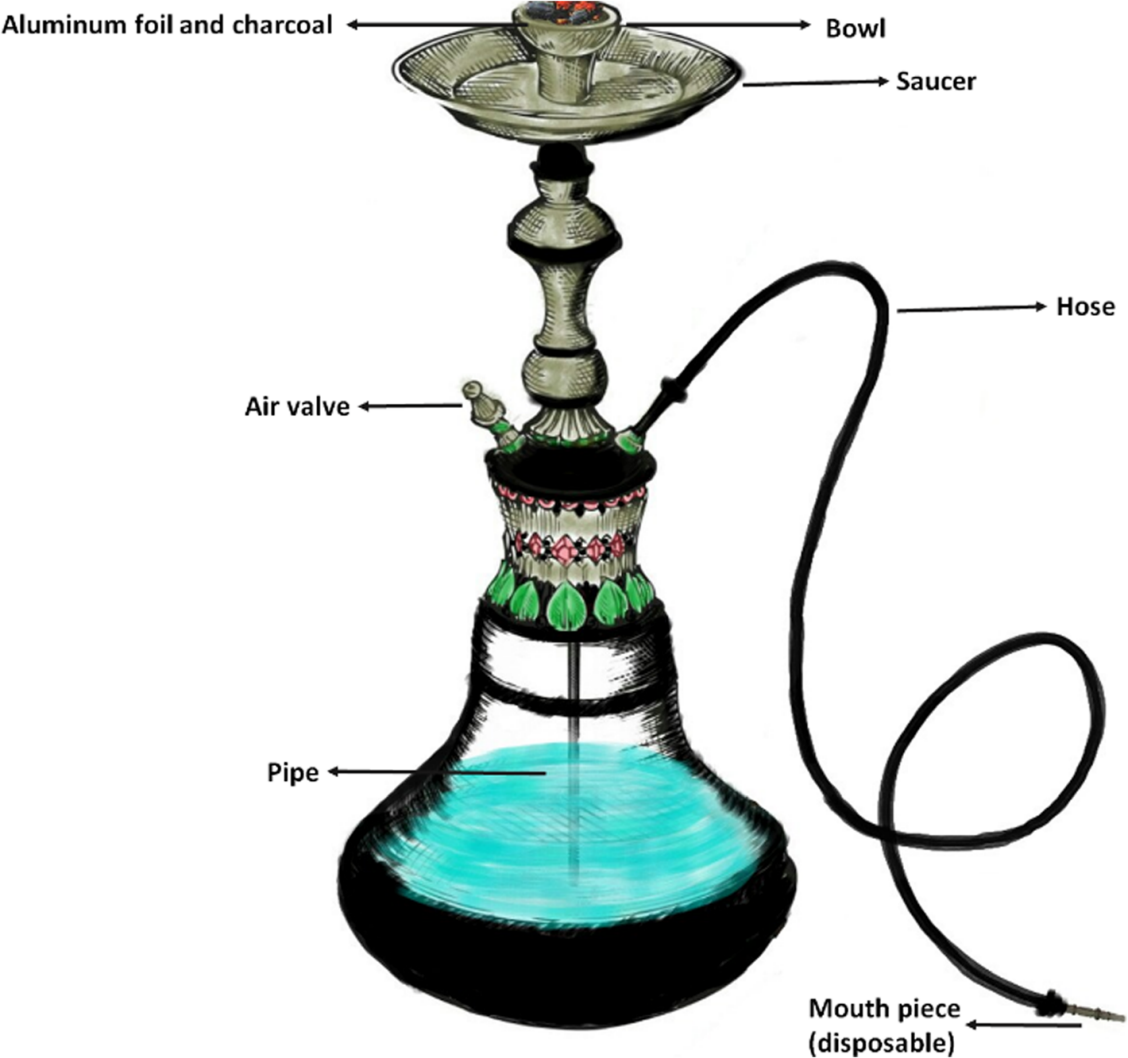
A typical hookah apparatus

The hookah or hookah apparatus is composed of an upper and lower compartment connected by a pipe (Fig. 2). Briefly, the top consists of a bowl where tobacco or molasses are placed then covered with perforated aluminum foil above which burning charcoal is placed. On the bottom of the apparatus resides a water jar covered by a gasket, protruding a hose and a release valve (used for clearing out stagnate smoke) [1–3]. A detailed description of hookah components is provided in Table 1.

**Table 1:** Hookah apparatus components and their use

Component	Usage/properties
Apparatus [1–4]	
Water jar (base)	Glass container that is filled with water; however, other liquids might be used (milk, fruit juices, or even alcohol).
Pipe (body, stem)	Metallic tube extending from the bowl to the base (partially immersed in water).
Bowl (head)	Carries (10–20 g) of tobacco.
Saucer	Holds charcoal ashes.
Accessories [3]	
Filter	A device that can be placed on the tip of the hose, marketed with claims that it reduces exposure to nicotine and tar.
Mouthpiece (extension)	A short plastic tip fitted into the hose as an extension to it.
Aluminum foil	Used as a membrane between tobacco (beneath it) and the charcoal (above it).
Consumables [3, 4]	
Charcoal	Special type of charcoal round pellets that is easily lit [212] (made from wood, coconut shell, or other material).
Tobacco	Marketed in two forms: moassel (fruit-flavored tobacco, pliable, and moist) and ajami (unflavored tobacco). Steam stones (heat-treated materials soaked with glycerin) are new “healthy” tobacco alternatives which are released [213].

This provides an overview of the various components of a hookah apparatus, as well as their use

Even though hookah retains some of its features globally (presence of liquid through which smoke passes), there is immense variability in the consumables, sizes, and materials used to manufacture apparatus parts, due to personal preferences and cultural/regional differences [4, 5]. It is noteworthy that manufacturing material variability may influence the levels of smoke/chemicals exposure. Indeed, one study reported that differences in pipe material affected the levels of carbon monoxide (CO) exposure, correlating the non-porous plastic hose with higher yields of CO compared to the more porous leather hose [6]. The same theory could be applied to yields of other chemicals, especially nicotine. Hence, hookah effects may be under/overestimated in some studies, so future research should take into consideration such variations to make results more relevant.

### Hookah tobacco

There are three commonly used types of hookah tobacco, Mouassal, Jurak, and Tumbak, each contains different ingredients. In brief, Mouassal which is an Arabic translation for “honeyed” contains 30% tobacco and around 70% honey/sugarcane as well as glycerol and flavors [7]. Hadidi and Mohammed [8] estimated that the nicotine contents of Mouassal is about 3.4 mg/g. Jurak, on the other hand, contains tobacco, sugarcane, and around 20% other spices and dried fruits [8]. Jurak is commonly used in the Middle East and Gulf region. Finally, Tumbak, which is used mainly in Asia, is a pure form of unflavored tobacco leaves (Ajami) smoked with charcoal.

### Hookah tobacco flavors

In the USA, there are different flavors used in the hookah tobacco with the most popular being the fruit flavors [9]. Similarly, among university students, fruit flavored tobacco was preferred to unflavored ones [10]. Among US women, candy/sweet and menthol are the second and third preferred flavors, respectively, with fruit flavors still the number one choice [11]. Other flavors include chocolate, clove/spice, alcohol, and other beverages [9]. This suggests that flavored tobacco plays a major role as a “motivator” for using hookah, which provides the user with the pleasant taste and smell.

### Hookah preparation and mechanism of action

The user or the person preparing the hookah (Fig. 2) starts by loading the tobacco into the bowl before wrapping the head with aluminum foil and then perforating the foil by using a screen pincher or toothpick. After that, the “ignited” charcoal is placed on the top perforated foil to initiate the tobacco heating process [12]. During inhalation, charcoal-heated air passes through the pierced aluminum foil and through the tobacco down the pipe and towards the water. After “bubbling” through the water, the cooled smoke reaches the surface and is drawn through the hose and is inhaled [3, 13]. Taken together, hookah smoking seems to have a complex puffing behavior when compared to conventional cigarette smoking.

### Puffing topography

Both cigarette- and hookah-smoking topography serves as an indirect measure of smoke and chemical exposure [12, 14]. In comparison to cigarettes, hookah puffing is more variable including total puffing time, number of puffs, and total smoke inhaled, all being affected by the nicotine content of tobacco, the presence of flavors, the personal preferences, and the social setting of the vaping session [12, 15, 16]. Regarding total puffing time, hookah use takes significantly longer periods (30–90 min/session) [12, 17–20] in comparison to cigarette smoking (averages 5–6 min) [12, 21]. Furthermore, number of puffs, mean puff duration, puff volume, and inter-puff intervals were higher in hookah [22–24], in contrast to conventional smoking [25, 26]. The longer sessions of hookah smoking could explain the increase in number of puffs/session. Also, the “humid” nature of the hookah smoke makes it more pleasant than the dry cigarette smoke and facilitates higher volume uptake [15, 16, 27]. Importantly, this higher humidity of the smoke and its cooled down nature facilitates deeper inhalation potentially increasing the side effects of using hookah [2]. Given the behavioral complexity of hookah use/smoking, further examination of smoking patterns is warranted in order to accurately estimate users’ exposure to harmful chemicals.

## Reasons and prevalence of hookah use

As mentioned before, hookah became widely popular, with its use accelerating rapidly especially among youth and women [28, 29]. Thus, understanding the reasons/patterns of use will aid in developing strategies to better control hookah use. Although there are ample justifications in the literature for hookah use, in this section, we will include the most commonly reported, in addition to the prevalence of hookah.

### Reasons of use

Many factors seem to have promoted hookahs’ spread/use, including but are not limited to perception of “no/less harm” of hookah, social acceptance/less restrictions, accessibility, use of flavored aromatic tobacco, curiosity, peer pressure, fashion, higher socioeconomic status, and need for amusement [28, 30–36]. One factor that drastically contributed to the increased hookah use (similarly to e-cigarette use) is the misperception about the health risks. Majority of users believe in the “no or less harm” of hookah compared to cigarettes; this particular belief could be connected with the myths of intermittent use of hookah that reduces harm compared to constant use of cigarettes [37], the passage of smoke through water would filter it, and “the less” addictive nature of hookah [2]. Some users argue that they do not inhale the smoke (keep it in the mouth cavity), therefore protecting themselves from nicotine absorption/addictive effects. However, nicotine could be easily absorbed through the mucosal lining of oral cavity [2]. Furthermore, receiving the “positive” attributes of hookah such as socializing, relaxing, and the good taste/smell of the smoke seems to encourage and maintain hookah use [9, 38]. To this end, a recent study conducted on social media (Twitter) found that social events and flavors were among the common contexts and experiences associated with Twitter discussions about hookah (2017–2018) [39]. Finally, one study demonstrated that participants preferred flavored hookah because the “sweet” flavored smoke smell is not viewed as offensive as cigarette smoke [36], which supports the conclusion that flavor plays an important role in promoting hookah use. Thus, clearly, there should be more emphasis on research studies that examine hookah health risks, which would inform campaigns for educating the public on the myths and the negative health effects associated with hookah use.

### Prevalence

**Fig. 3:**
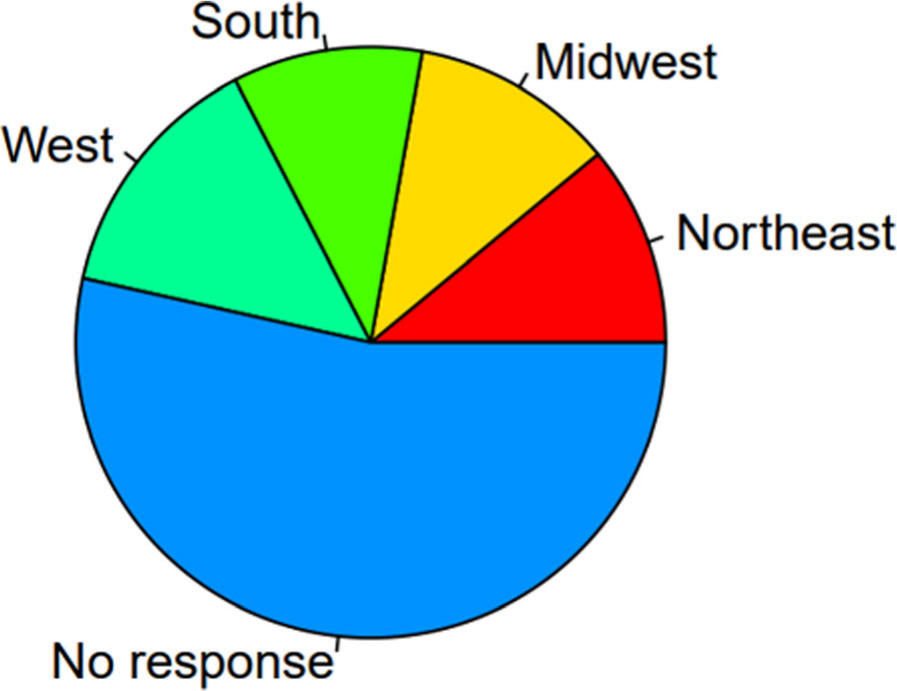
Distribution of hookah “ever use” in the USA, from the National Adult Tobacco Survey (NATS) 2012–2013 (*n* = 60,192) [47]

Worldwide, it is estimated that 100 million people use hookah on a daily bases [40]. Back in 2011 “current hookah use” among adults age > 18 years was 15% in Lebanon, 9–12% in Syria, 4–12% in Arabic gulf countries, 6% in Pakistan [41], and 30% in Jordan [42], whereas in Iran, it was found that more non-smokers transition to tobacco use including waterpipe/hookah [43]. Comparable to those levels, US adults “current use-smoking waterpipe on at least 1 day within the past 30 days” was 9.8% and “ever use-smoking waterpipe at any point in lifetime” was 1.5% between 2009 and 2010 [44] reaching levels of 12.3% and 3.3%, respectively, by 2012–2013, reflecting a gross increase within the US population [45]. Furthermore, while the majority of US hookah users are also tobacco smokers, a significant portion of hookah users are non-smokers [35, 38]. In this connection, differences between rural and urban US in smoking hookah were also examined, and the results illustrated more prevalence of hookah use in urban areas in comparison to rural areas [46]. This difference could be attributed to the sociocultural and economic factors linked to living in urban areas. Besides that, the distribution among the states/regions was found to be variable [47] (Fig. 3), with west states having higher prevalence compared to south states, in particular five states, namely Arizona, California, Colorado, the District of Columbia, and Nevada, had high rates of both current hookah use ( ≥ 5%) and ever use (≥ 15%) [45]. It is difficult to determine what might be the cause behind this increase in the west, but potentially, it might derive—in part—from the higher population of Arab Americans within these regions. This notion is supported by Arab Americans having the highest rates among all racial/ethnic groups of adults identifying themselves as non-Hispanic “Other” [44]. Regardless, conducting research on hookah environment characteristics such as the number of hookah bars, methods of advertising, and social behaviors could serve as the first step in further understanding the increasing prevalence and popularity of hookah.

Hookah use among US youth population in schools was under scrutiny in many studies. For instance, one study surveyed a representative sample of 6th–12th grade students for hookah use, and the results showed that 10.5% reported smoking hookah [48]. Another study documented that hookah “ever use” among middle and high school students in USA included 6.8–15% of the students [31]. These results are remarkable as they show that as young as sixth grade (vulnerable population) can be a user of hookah, and potentially exposed to all associated health risks. According to the same study, household hookah users and easy access are the main motivation to use hookah in such young age [48]. Interestingly, Arab Americans had higher percentage of both “current and ever use” compared to non-Arabs, which indicates a strong influence for cultural background on hookah use [49].

College students’ prevalence of hookah use was 9.6% for “current use” and 22.9% for “ever use” between 2008 and 2009, whereas it increased to 28.4% for “past year” use and 46.4% for “ever use” in 2013 [50]. Such an alarming increase may be in part due to the belief that hookah use is less harmful/addictive with higher social approval in comparison to tobacco [51, 52]. Notably, this belief is the major reason many adults use hookah, in addition to viewing it as a good way of socializing, and the belief that it helps quitting cigarettes, as well as being relatively cheaper than smoking cigarettes [34]. Another drastic and more concerning increase of 5.3% from 2011 to 2014 in hookah popularity was reported among adolescents in USA. This is especially troublesome as adolescents continue to be exposed to harmful tobacco product constituents, in particular nicotine that might interfere with brain development, cause addiction, and might lead to sustained future tobacco use [53]. Another recent representative sample of young adults aged 15–24 years old revealed that hookah use was 14.7% among males and 10.2% among females [54]. This increase in use could be attributed to the perception of fewer negative consequences of hookah smoking compared with cigarette smoking and the social norms regarding its acceptability among this population [55–57].

Importantly, developing new perhaps “more rigid” policies to regulate hookah use is not only needed in youth but must also expand to control use during pregnancy. This is rather a public health priority given hookah’s prevalence (12.4%) [58], coupled with the relatively “high” passive exposure to hookah smoke (32.8%) during gestation [59], which in turn leads to the involuntary exposure of innocent fetuses to hookah, and subjecting them to hookah’s potential harmful effects [60].

Regardless of the variability in hookah prevalence among the various populations, clearly, there is an overall drastic increase in its use over a short period. Longitudinal studies should further help in understanding and evaluating use trends, sociodemographic characteristics, and health risks in various populations being exposed to/using hookah, which would ultimately shape new “more strict” policies, especially those governing use among highly vulnerable populations, including pregnant females and youth.

## Toxicants and air quality

### Toxicological profile

Even though hookah has been present for a millennium, far less studies have examined its chemical constituents/air quality relative to cigarettes. With tobacco being the main source of smoke in both hookah and cigarettes, hookah users are exposed to many of the same toxic compounds/by-products as cigarette users but at dramatically higher levels, which might in fact produce worsened health effects in users [23]. Consequently, it is important to evaluate the major compounds expelled from hookah vape, in order to aid in evaluating both acute and chronic health outcomes.

**Fig. 4:**
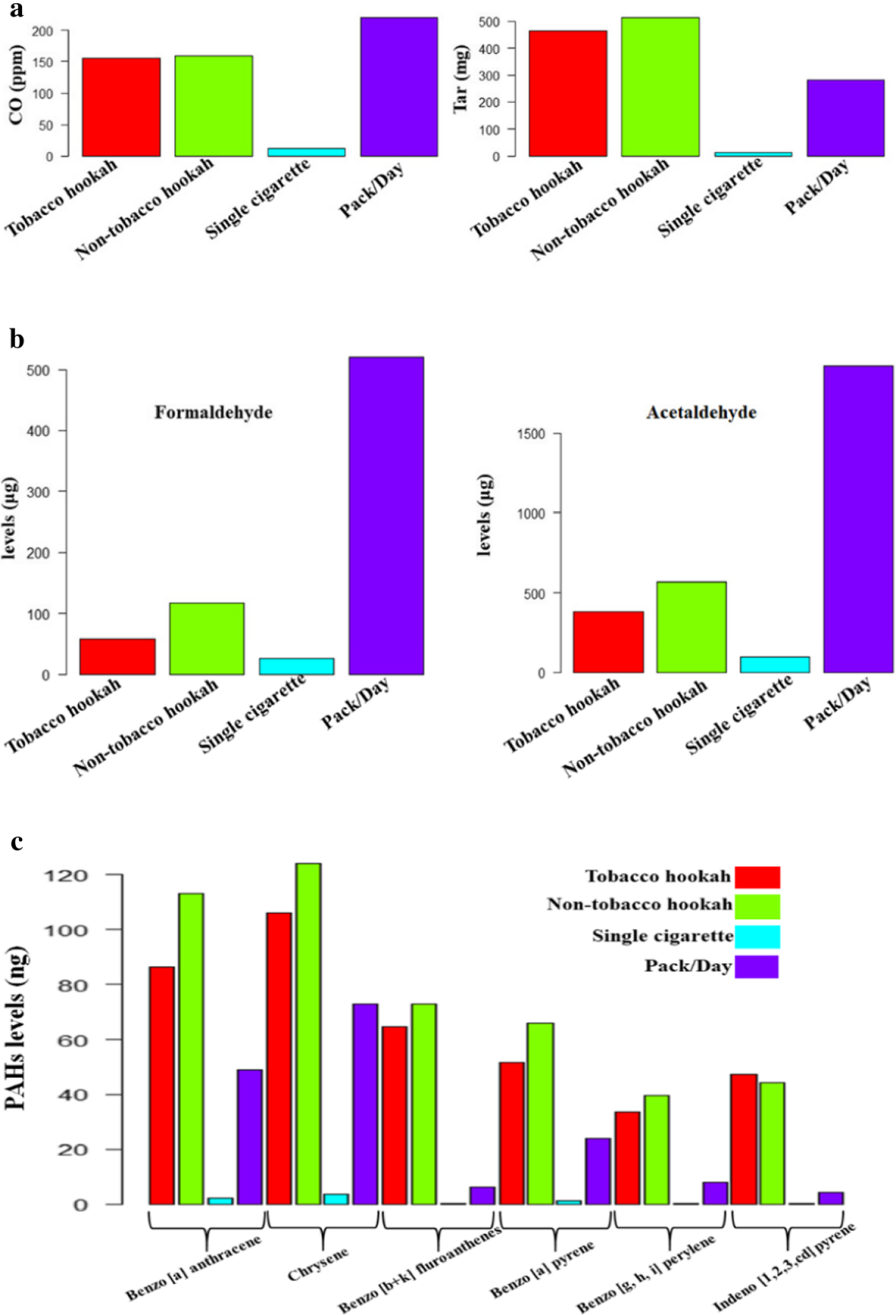
Comparison of the levels of some toxicant expelled in both hookah and tobacco smoke. Levels of the toxicants (**a**) tar, CO, (**b**) carbonyl compounds (formaldehyde and acetaldehyde), and (**c**) certain PAHs are indicated. Levels of toxicants in pack/day are extrapolated by multiplying the levels in one cigarette by 20 [71–74]

Several toxicants have been found in mainstream hookah smoke including nicotine [27, 61–64], carbon monoxide [27, 63], carcinogenic polycyclic aromatic hydrocarbons (PAHs) [27, 61, 63–65], aromatic amines [63], aldehydes [64, 66], furanic and phenolic compounds [67, 68], tar [19, 61], particulate matter [69], heavy metals [19], and ammonia [70]. It is noteworthy that the amounts of these toxicants might be higher/lower in hookah compared with cigarette smoke (per cigarette/and per pack/day) due to different heating process and charcoal combustion [71–74] (Fig. 4).

Nicotine—the major source of tobacco addiction—content in hookah is extremely variable as it depends on the type of tobacco used. Consequently, the amount uptaken by the user depends on hookah use characteristics that are adjusted depending on nicotine levels in tobacco used in order to deliver desired doses [62]. Similar to cigarette smoking, plasma nicotine levels were found to be increased in hookah users, indicating systematic delivery of nicotine. However, these levels were much higher compared to cigarette users, which could be explained by longer “hookah” sessions with a higher puff number/volume [23, 75]. Likewise, plasma carboxyhemoglobin levels in hookah users exceeded those of cigarette smokers’ levels [61], indicating the presence/inhalation of carbon monoxide (CO) during hookah use. This is because CO displaces O_2_ from hemoglobin forming carboxyhemoglobin (CO affinity for hemoglobin is 200 times that of O_2_) and shifting the oxygen dissociation curve to the left, thereby causing hypoxia and impairment of cellular respiration [76]. Notably, hookah use was linked to several cases of CO poisoning [77–81]. Furthermore, NNAL—a metabolite formed after 4-(methylnitrosamino)-1-(3-pyridyl)-1-butanone (NNK) enters the body and a well known carcinogen—urinary levels increased markedly after hookah use, indicating the presence of tobacco-specific nitrosamines in hookah smoke [63, 82]. Other well-known carcinogens/potential carcinogens are polycyclic aromatic hydrocarbons (PAHs), and they have been quantified in hookah smoke, in particular 16 of these compounds have been found [65]. Moreover, in contrast to single cigarette smoking, nicotine-free dry particulate matter (TAR) from a single 45 min hookah session reached a level of 802 mg, which represents 36.5 folds higher than that in a cigarette. This should in turn diminish marketing fads of hookah containing 0% tar [83]. Of note, levels of tar delivered vary from session to session, reaching up to 100 folds in some cases of longer hookah smoking sessions [84].

Importantly, inhaled volumes are concerning because they deliver high amounts of hazardous chemicals/session compared to cigarettes. For instance, aldehydes, such as acrolein, induce cardiopulmonary toxicity [85, 86], are potentially carcinogenic [87, 88], and are prothrombotic [89]. Furthermore, the PAHs are carcinogenic [90], whereas carbon monoxide induces cardiovascular disease [91], and nicotine is known for its addictive nature [92]. As for ammonia, which is a strong respiratory irritant, its levels should be measured as part of the assessment of the hookah toxicant profile, and this could be achieved using a simple colorimetric method as was recently described for determining ammonia levels in tobacco fillers and sidestream smoke in different tobacco brands [93].

Based on the aforementioned considerations, hookah use poses as many or even higher risks for the smoker as cigarette smoking. Besides tar, hookah bears additional risks such as an uptake of addictive and carcinogenic/ potentially carcinogenic chemicals, which stands in contrast to the “massive” advertisement of them as a “healthy” smoking product.

### Air quality and passive exposure

**Fig. 5:**
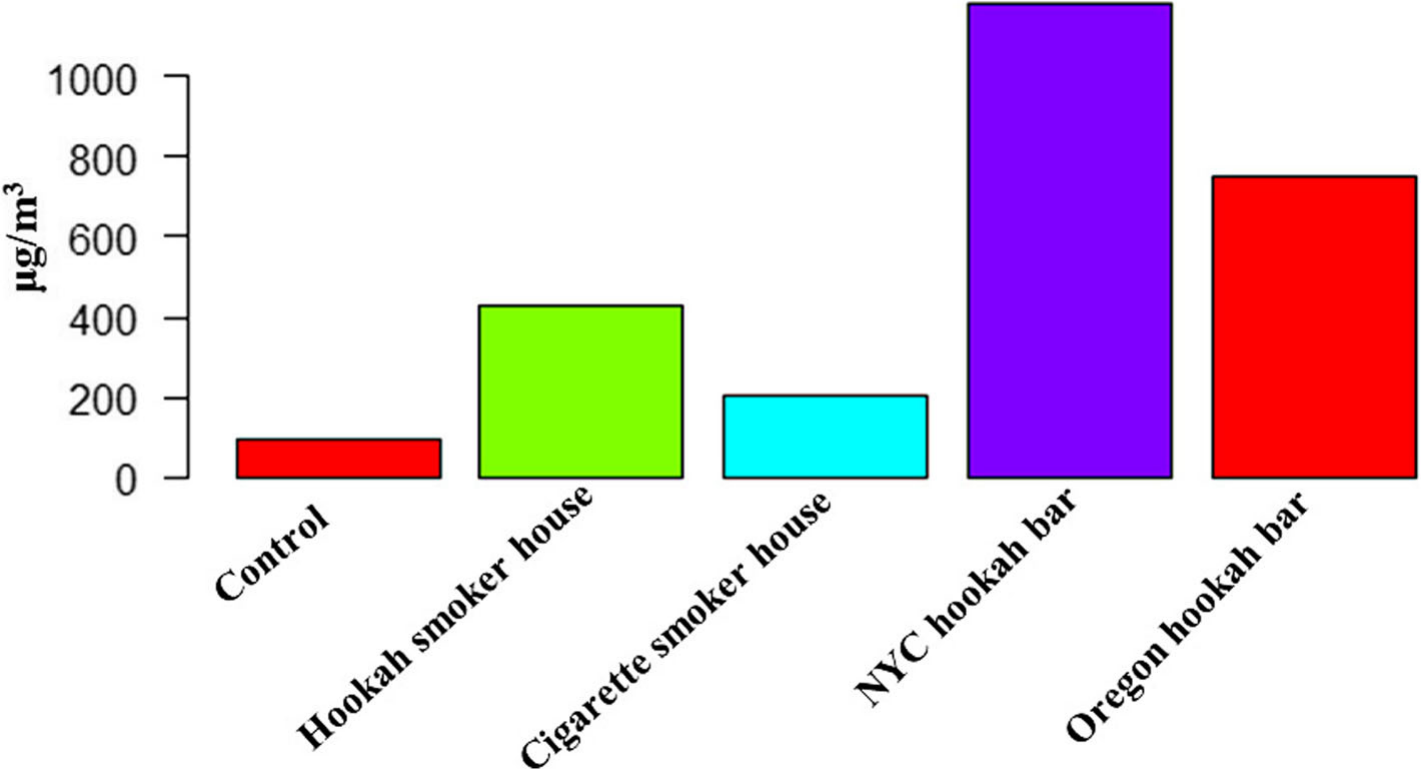
Comparison between PM2.5 levels in houses of hookah users, cigarette users, and bars in two states. NYC hookah bars (*n* = 8). Oregon hookah bars (*n* = 10) [94, 96, 98]

Ambient concentrations of particulate matter are often used to assess pollution levels from tobacco smoke [94]. Cigarette smoking expels high levels of particulate matter in bars, exposing both customers and employees passively to hazardous levels of pollutants [95]. In similar fashion, the examined air quality in hookah lounges ranged from “unhealthy” to “hazardous” by the Environmental Protection Agency (EPA), containing high concentrations of particulate matter [94, 96, 97]. Such air quality poses health risks, especially among those with existing pulmonary and cardiovascular disease, and presents potential health hazard for workers who can be exposed to secondhand hookah/smoke on a daily basis and for prolonged periods of time [94]. As expected, low air quality was reported in houses of hookah users, and interestingly, toxicant levels were greater than those in cigarette smoking homes, yet less than those in lounges/bars (Fig. 5; [94, 96, 98]), which is probably due to lower numbers of hookah apparatuses used [98]. Nonetheless, such low air quality exposes non-users within the household to hazardous materials and puts them under increased risk of disease, especially if they are highly vulnerable (with chronic disease, children, and pregnant women). Consequently, there is a marked need for further research, policies, and better air quality monitoring to improve the indoor air quality in order to reduce passive exposure and its negative health consequences.

## Hookah health hazards

While hookah users and those in close proximity are exposed to many of the potentially dangerous toxicants at one time, the health risks associated with its use continue to be under debate. This is—in part—attributed to the fact that the composition of tobacco smoke in hookah and its puffing patterns are variable and not well standardized. Nonetheless, several studies have provided evidence of health impairments that are associated with hookah use.

Theoretically, sharing the mouthpiece during hookah group smoking can be a probable source of transmission of pathogens such as viruses, bacteria, and fungi. For instance, a study reported a potential risk for transmission of communicable diseases such as hepatitis C when sharing the mouthpiece between users with bleeding gum [99]. Also, the “non-hygienic” conditions of the hose and water in the hookah apparatus could also increase mycobacteria growth, which can result in spreading/transmission of tuberculosis [100, 101]. Other studies have also linked hookah to transmission of *Helicobacter pylori* (main cause of peptic ulcer) and *Aspergillus* spores (cause of pneumonia in immunocompromised patients) [102, 103]. Moreover, 48 bacterial isolates were detected from hookah hoses, and among them were virulent as well as antibiotic-resistant strains [104]. Furthermore, using hookah was linked to developing periodontal diseases in similar magnitude to cigarettes [105] as well as documented alteration in oral microbial flora [106].

Similar to cigarette smoking, hookah use is also linked to a harmful impact on the pulmonary system. Thus, hookah users complain of symptoms such as wheezing, cough, sputum, and shortness of breath [107–109]. Furthermore, hookah significantly decreases pulmonary function parameters, including FEV1, FEV1/FVC ratio, and FEF, as well as the levels of FeNO [110–112]. FeNO is an essential marker of eosinophilic airway inflammation, and reduction in its levels may be due to rapid conversion of nitric oxide to peroxynitrite by reactive oxygen and nitrogen species or downregulation of nitric oxide synthase [113, 114]. Also, hookah users had lower lung diffusing capacity and elevated levels of apoptotic endothelial cell microparticles [109]. Hookah exposure induced a significant elevation of macrophages, lymphocytes, and neutrophils in broncho-alveolar lavage fluid and altered the levels of several cytokines. Thus, the levels of the pro-inflammatory cytokines TNFα, IL-1 β, IL-6, IL-12, and IL-13 were elevated, whereas the levels of the anti-inflammatory cytokine IL-10 were reduced, in the lungs of exposed mice [115, 116]. It also increased catalase activity in the lung and resulted in changes in the level and mRNA of major matrix metalloproteinases (MMP-1, MMP-9, and MMP-12), confirming pulmonary damage associated with hookah use [117, 118]. Moreover, chronic (4 months) exposure to hookah smoke in mice resulted in significant increases in alveolar destructive index and mean linear intercept contributing to the chronic obstructive pulmonary disease picture in these animals [119].

Importantly, hookah hazards are not limited only to oral/pulmonary systems. To this end, in a population-based study, hookah use was associated with metabolic syndrome development. Thus, hookah users had significantly higher incidence of hypertriglyceridemia and hyperglycemia, as well as hypertension and abdominal obesity, which was observed after controlling for age, sex, social class, and area of residence [120]. All of these “disorders” increase the risk of metabolic syndrome development, which is a major risk factor for developing thrombosis [121].

Unfortunately and as stated before, the number of hookah users among the vulnerable populations of pregnant females and adolescents is increasing. In fact, pregnant females still use hookah during pregnancy, regardless of its reported hazards. While may vary based on levels of use/exposure, a reduction of weight of the newborn (at least 100 g) in females using hookah once/day during pregnancy was evident. Moreover, the risk of delivering low birth babies tripled, in addition to reported neonatal respiratory distress that is linked to hookah use during the first trimester [122, 123]. In addition to reduction in birth weight, it was reported that hookah smoking during pregnancy contributes to a significant reduction in newborns’ other anthropometric measurements such as mean newborn length and mean newborn head circumference [124]. In a rat exposure model, hookah smoke exposure was shown to be associated with low birth weight, increased neonatal death rate, and lower growth rate among offspring [125]. Additionally, prenatal exposure to hookah smoke in a murine model of asthma in adult mice offspring also induced airway inflammation and adversely affected lung function [126]. In utero exposure to hookah tobacco smoke in rats resulted in impaired memory and decreased brain-derived neurotrophic factor in hippocampus of adult male offspring rats [127].

A study of hookah use among 7th–10th grade students indicated that it may impair adolescent brain development, given that it reduces the levels of the brain-derived neutrophic factor (BDNF) [128], which is essential for cognition and behavior [128]. A relatively recent study also reported a reduction in BDNF serum levels in students reflecting a possibility of systematic adverse health alterations in adolescence, coupled with behavioral changes (low attention and aggression) [129]. Moreover, hookah tobacco smoke exposure in rats induced short- and long-term spatial memory impairment [130], which was associated with reduced hippocampal levels of major oxidative stress biomarkers and oxidative capacity enzymes [131, 132].

With respect to carcinogenicity of hookah, it was reported that carcinoembryonic antigen (CEA) levels were higher in hookah smokers, in comparison to non-smokers, yet not as high as in cigarette smokers [133]. Thus, prolonged or heavy use of hookah may induce risk of tumor development, especially in oral cavity and esophagus, which further argues against the notion that hookah has “no/less harm” if not inhaled “kept in mouth.” Indeed, incidence of benign lesions of the vocal cords was linked to the presence of cysts in 4.8% of hookah users, which was similar to cigarette smokers [134]. Furthermore, three case-control studies reported a link between the risk of esophageal cancer and hookah use, with the risk increasing with cumulative use, higher frequency, and the duration of use [135–137]. Additionally, using hookah was linked to an average of six folds higher risk of lung cancer [138–141]. Moreover, it was reported that hookah use may increase the risk of gastric cancer by threefold, albeit the mechanism remains unknown [142]. In addition, hookah smoking was shown to be genotoxic, leading to DNA damage in lymphocytes, where the magnitude of its genotoxicity was higher than that induced by cigarette smoking [143, 144]. Exposure to hookah smoking resulted in elevated plasma and saliva levels of toxic metals, namely cadmium, copper, and zinc [145], which could contribute to its long-term carcinogenicity. Finally, another study (analyzed data collected from 152 academic institutions; *n* = 100,891 students) found moderate association between hookah smoking and mental health variables, such as depression, anxiety, and addictive disorders, among college students [146]. These findings provide evidence that hookah disrupts not only the “physical” health of the user, but also their mental state.

**Fig. 6:**
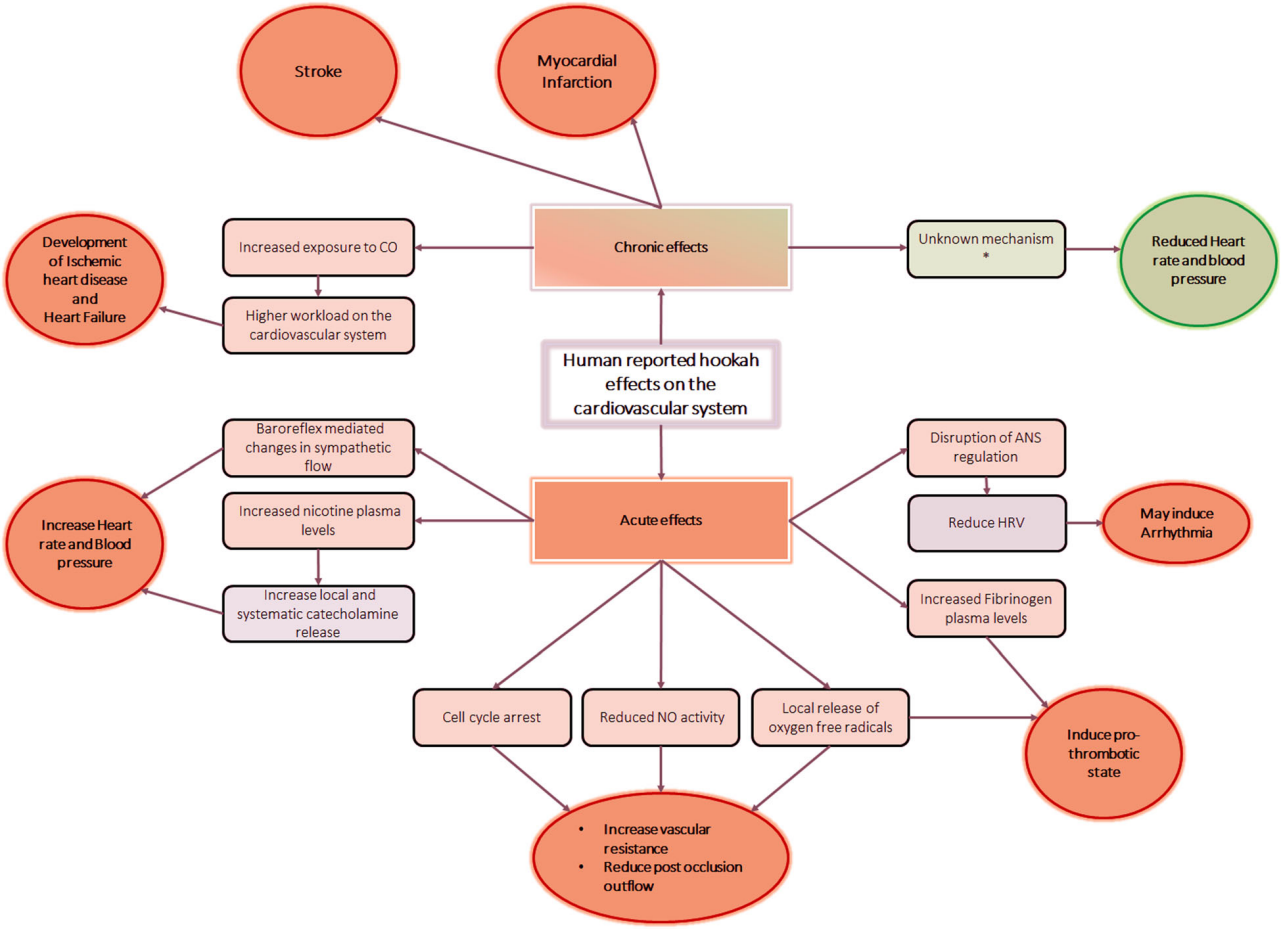
Cardiovascular effects and their underlying mechanisms. These data are compiled from what is reported in clinical studies. *In a study performed in adolescents, the reduced heart rate and blood pressure may be linked to the “abstinence of vaping” for 12 h prior to testing [195]

It is noteworthy that many of the aforementioned studies had limitations, for example, no control over use of other forms of tobacco and lack adjustments of the cofounding factors in some case studies, as well as limited assessment of gender and age as cofounders. Nonetheless and taken together, there is sufficient evidence in support of the association of hookah use with negative human health outcomes. Considering the cardiovascular system sensitivity and its non-linear dose-response/toxicity relationship with “smoke,” we sought to review the literature with regard to the effects of hookah on the cardiovascular system (see Fig. 6), both human and animal studies.

## Cardiovascular effects of hookah use

The detrimental (acute and chronic) effects of tobacco smoking on the cardiovascular system are well established [147–152]. Thus, cigarette smoking predisposes to cardiovascular events and contributes significantly to cardiovascular-related mortality and morbidity, being responsible for up to 30% of heart disease-related deaths in the USA each year [149, 153]. Importantly, hookah has overlapping toxicant/chemical profile to conventional cigarettes. In light of that, it has been shown that hookah smoke effects on the cardiovascular system are comparable to those of conventional cigarettes. It is noteworthy that a recent meta-analysis reported an odds ratio of association between hookah tobacco smoking and heart disease of 1.67 (95% CI = 1.25, 2.24) [154], which supports the notion that hookah is indeed detrimental to the cardiovascular system.

### Clinical studies

#### Acute cardiovascular effects

Acute effects of conventional smoking, such as increased blood pressure, heart rate, and vascular resistance, have been known for decades [155–160]. As with tobacco smoking, the instantaneous effects of hookah use include higher systolic and mean arterial blood pressure, as well as elevated heart rate (HR) [17, 23, 161–166]. It is noteworthy that in one cohort study, HR elevation has exceeded 50 bpm in 6.4% of participants and reached higher than 200 bpm in 3.6% of participants after only a 30-min hookah session [167]. These effects have been attributed, in part, to the baroreflex mechanism impairment [164] or to elevated nicotine plasma level. The latter exhibits adrenergic effects that will enhance local and systemic catecholamine release [15, 18, 163, 168–170]. Supporting the latter notion, mean post-hookah-smoking HR elevation was doubled in participants using nicotine-containing hookah in comparison to nicotine-free hookah smokers [171]. Additionally, in a two double-blind study, “placebo” had no effect on HR, while hookah-smoking increased it significantly [15]. Another study reported that changes in the cardiovascular central and peripheral components occur immediately after hookah smoking and include increases in HR, blood pressure, and after occlusion vascular resistance, whereas after occlusion blood flow and outflow were decreased [172]. The cardiovascular changes were shown to be exacerbated among individuals with low habitual physical activity and physical fitness levels [173]. More recently, it has been reported that adolescents smoking hookah had significantly lower vascular endothelium growth factor (VEGF) levels [174], which might adversely affect vascular growth and function in this population.

Acute use of hookah also induced changes in the peripheral vascular system in similar fashion to cigarette smoking, such that it increased vascular resistance and reduced post-occlusion blood flow. This could be linked to local release/synthesis of oxygen-derived free radicals, cell cycle arrest, and decreased in NO activity [175–178]. In a manner comparable to cigarette smoking, short-term hookah use significantly impaired flow-mediated dilation (FMD), which indicates endothelial dysfunction, but hookah was a weaker predictor for high risk profile [177]. Furthermore, it was reported that short-term hookah use (both tobacco-based and tobacco-free products) disrupts the autonomic nervous system regulation on the cardiac cycle, thereby causing a reduction in HRvariability, which—in turn—might aggravate the risk of coronary artery disease development [18]. Moreover, a significant increase in TXB_2_ levels, a metabolite of the biologically active TXA_2_, and an index of oxidative injury were reported after a single hookah smoking session [179]. This increase in TXB_2_ levels would suggest an increase in platelet activity [180]. Importantly, it has been shown that an increase in platelet activity plays a major role in the pathogenesis of acute myocardial infarction (MI) [181] and acute stroke [182–184]. Therefore, it is not surprising to see a link between hookah smoking and acute MI in young adults [185], and among patient undergoing cardiac catheterization [186]. However, no data exist yet on the association between hookah smoking and acute stroke [187]. Interestingly, and contrary to the hypothesis that hookah decreases myocardial blood flow because of the charcoal combustion nanoparticles (vasoconstrictor), a study found that hookah use acutely increased myocardial blood flow. This is thought to be due to cardiac β-adrenergic stimulation as physiological response to increased myocardial work and oxygen demand [188]. In light of the aforementioned evidence, it is clear that even short-term use of hookah disrupts normal cardiovascular function, as repetitive short-term hookah exposure may be the triggering point of causal chain of reactions ultimately leading to the chronic effects. Nonetheless, more research should be done to evaluate hookah’s effects, which will guide awareness campaigns regarding its negative health outcomes, including those that result from short-term use/exposure.

#### Cardiovascular effects of chronic use

With regard to the adverse cardiovascular effects associated with longer-term of hookah use, they are comparable to those associated with cigarette smoking. In this connection, a link between chronic use of hookah and coronary artery disease (CAD) development has been shown, with the frequency and duration of exposure being critical risk factors to CAD. In fact, individuals with more than 40 years of hookah smoking had three times more risk of having severe stenosis than non-smokers [189]. Additionally, cardiovascular disease development such as ischemic heart disease (IHD) and heart failure has been associated with heavy hookah smoking [190]. These outcomes could be explained by the continuous stress placed on the cardiovascular system as result of exposure to high amounts of CO [191]. Furthermore, death due to IHD was 1.96 folds in ever hookah smokers with higher daily intensity of hookah smoking than never users [192]. Hookah smoking was also associated with severe coronary artery disease, which was dependent on the duration/frequency of hookah smoking [193]. In accordance with the latter data, dose-response relationship between hookah-years and percent stenosis was also established [189]. Furthermore, risk of MI and stroke death was significantly increased with hookah smoking. Finally, higher fibrinogen plasma levels were reported in long-term hookah smokers (> 10 years), which elevate the incidence of pro-thrombotic/atherosclerotic events [194] and might explain in part the higher risks of stroke and MI linked with chronic hookah smoking.

Notably and interestingly, a recent cross-sectional study aimed to examine the relationship between chronic hookah smoking and cardiovascular hemodynamics in adolescents found a reduction in both BP and HR of adolescent hookah smokers versus non-smokers, which is in contrast to previously reported results in adults. This might be explained by the “abstinence of” hookah smoking for 12 h prior to testing, thereby reducing nicotine levels drastically in the system, which would impact neuro-hormonal regulation (reduced cortisol and sympathetic activity). Nonetheless, the exact mechanism underlying such outcome is still unclear but warrants investigation [195].

Together, hookah use has been associated with many cardiovascular effects that influence or contribute to the decline of the overall health status of members of our communities. Unfortunately, despite studies documenting cardiovascular disease risks associated with hookah, people continue to assume that it is safer than cigarettes, mainly due to being unaware of its negative health effects. Thus, hookah awareness/control should be more robust and systematic, but more importantly, further studies need to be conducted to better understand its negative health consequences and the mechanisms inducing such effects.

#### Animal studies

Acute and chronic exposures to hookah smoke resulted in significant changes in kidney function biomarkers such as creatinine and blood urea nitrogen, in mice. This was associated with reduction in antioxidant enzymes and biomarkers including superoxide dismutase for acute and chronic hookah smoke exposures, and catalase, glutathione peroxidase, and thiobarbituric acid reactive substances for chronic exposure [196]. Acute and chronic exposure of mice to hookah corroborates with the clinical findings that suggest cardiovascular dysfunction. Thus, short-term nose-only exposure to mainstream hookah for 5 consecutive days induced a significant decrease in platelet numbers and amplified in vitro platelet aggregation indicating a prothrombotic state [197, 198]. Furthermore, cardiac inflammation with an increase in reactive oxygen species (ROS) was observed, which consequently caused an elevation in heart glutathione (GSH; an antioxidant) concentrations. This seems to indicate that an initial adaptive response that counterbalances the potentially damaging activity of ROS [197] is triggered. Interestingly, long-term nose-only exposure for 1 month caused a significant increase of ROS in the heart accompanied with decreased heart GSH concentrations in exposed mice, indicating depletion of the antioxidant, which increases heart tissue's vulnerability to oxygen free radical damage [199]. The increased cardiac vulnerability may explain the increased systolic blood pressure reported after long-term use, which was not seen post-short-term exposure [197, 199].

In summary, both clinical and animal studies have provided substantial evidence of a link between cardiovascular disease development and hookah (short- and long-term use). However, there remain some knowledge gaps; firstly, there is a lack of well-designed studies addressing the association between hookah use and cardiovascular diseases. Second, the pathophysiologic mechanisms underlying the cardiovascular adverse effect are not fully understood, and thus, studies to address these issues are not only warranted but also critical at this point.

## Emergence of novel hookahs (e-hookah)

Electronic nicotine delivery systems (ENDS) are battery-vaping devices that heat a liquid (e-liquid)—which may/may not be flavored and may/may not contain nicotine, thereby producing a vape that is inhaled by the user [200]. Multiple studies assessed electronic cigarettes’ (e-cigarettes) health effects which are still under debate, since they emerged in 2007 to US market [201], which is in contrast to the minimally studied electronic hookah (e-hookah) that debuted in 2014 across US markets [202]. For instance, e-cigarette use has been linked to increased health risks, including increase thrombosis risk [203] as well as throat and mouth irritation, respiratory tract irritation, and behavioral changes among others [153]. Whether e-hookah is similar to e-cigarettes in terms of exerting negative health effects is not known yet, as thus far, studies on e-hookah use/effects are limited. To this end, a recent pilot study aiming to clarify the differences (if present) between e-cigarette and e-hookah reported the following: (1) 94% of e-hookah are disposable compared to only 40% of e-cigarettes, (2) the majority of e-hookahs came with flavors compared to e-cigarettes, and (3) 91.7% of e-hookah were labeled “nicotine free” relative to 5.9% of e-cigarettes, which taken together supports the notion that the purpose of e-hookah is social/recreational in nature [202]. It is to be noted that despite the fact that most e-hookahs are labeled “nicotine free,” their safety is still unknown. Unfortunately, age restrictions on e-hookah packages apply only to 50% of the products [202], thus facilitating their purchase/use by minors. As for the use of e-liquids in e-hookah, that would be expected to produce the same toxicity as e-cigarettes. The evolving nature of these devices supports the notion that investigating their use patterns, purpose, prevalence, and potential health effects is crucial. Meanwhile, the public health experts should educate the public about the possible yet unknown health hazards of these products, whereas policy makers should limit their access to youth.

## Hookah regulations and policies

Based on the scientific evidence of the toxicity associated with tobacco use (passive/active), an increasing number of states has instituted/is instituting regulations to support/expand legislation of clear indoor air quality to include hookah [204, 205]. As mentioned before, hookah smoke may be associated with similar or even greater inhalation of toxicants in comparison to cigarettes. Since 2016, the FDA finalized a rule extending their control of all tobacco products, including hookah tobacco. FDA now regulates the manufacture, import, packaging, labeling, advertising, promotion, and sale, as well as distribution of hookah tobacco and of all hookah apparatus parts (except the accessories; lighters and tongs) [206]. However, some US legislations/policies controlling cigarettes do not similarly apply to hookah [31]. For instance, the “Prevent All Cigarette Trafficking Act” prevents US Postal Service to ship cigarettes but does not interfere with hookah shipping [207]. Additionally, nearly 90% of the largest US cities may allow hookah in bars via exemptions (whereas cigarettes are prohibited) [208]. Unfortunately, youth represent a large portion of the hookah user population, and their accessibility is facilitated through online ordering. To control such means of access, major credit card companies should ban online payments for hookah, as they did with cigarettes [209]. Clearly, policies and policy improvements are crucial, and research on hookah and its health effects would be helpful/important to health policy officials seeking to update/refine them.

## Conclusion

Tobacco is a preventable cause of morbidity and mortality worldwide. In recent years, hookah use increased mainly as an alternate tobacco smoking method, under the assumption of it being “less harmful” [210]. Lately, as also noted by the American Heart Association statement [211], hookah has been considered a global threat—in part—due to the high increase of its use, in addition to the deleterious effects it has on human body such as frequent respiratory infections, persistent cough, oral and esophageal cancer, and induction of a pro-inflammatory state. Regarding hookah’s cardiovascular toxicity, unlike in case of smoking, little is known about those associated with hookah. Nevertheless, and based on the current evidence, it is now known that hookah emits various potentially harmful and toxic chemicals, and therefore, it should not be considered a “healthy alternative” to smoking. In fact, in light of the greater volumes expelled from hookah/session, it is still under debate whether the levels of the toxicants it emits are lower/higher than traditional smoking/day. In this connection, recent studies have shown that the levels of hookah-emitted chemicals vary depending on multiple factors such as topography, experience, session length, and type of tobacco used during each session. Regardless whether hookah is as toxic or less toxic than cigarettes, its harm is evident to certain extent, and it can still extend to innocent/bystander non-smokers through passive exposure, including children, pregnant women, housekeeping workers, and people with pre-existing cardiovascular and other diseases. The widespread and increasing usage of hookah in the USA is concerning. Therefore, funding should be allocated/dedicated for future research on hookah, to examine its acute/long-term effects on the cardiovascular and other systems of both active and passive users, as well as provide mechanistic insights regarding its negative health effects. Collectively, these findings can be used in educational campaigns for the public, as well as in shaping policies for further evidence-based hookah control.

## Data Availability

There is no “original” or unpublished data in this review article. However, the corresponding authors will share the sources of the data upon request.

## Abbreviations

BDNF: Brain-derived neutrophic factor
CAD: Coronary artery disease
CEA: Carcinoembryonic antigen
CO: Carbon monoxide
DNA: Deoxyribonucleic acid
ENDS: Electronic nicotine delivery systems
EPA: Environmental Protection Agency
FDA: Food and Drug Administration
FEF: Forced expiratory flow
FeNO: Fractional exhaled nitric oxide
FEV: Forced expiratory volume
FMD: Flow-mediated dilation
FVC: Forced vital capacity
GSH: Glutathione
HR: Heart rate
IHD: Ischemic heart disease
IL: Interleukin
MI: Myocardial infarction
mRNA: Messenger RNA
NO: Nitric oxide
PAHs: Polycyclic aromatic hydrocarbons
RNA: Ribonucleic acid
ROS: Reactive oxygen species
TNF: Tumor necrosis factor
TXB_2_: Thromboxane B_2_
VEGF: Vascular endothelium growth factor
